# Combined Forced and Thermocreep Convection through a Long Horizontal Microchannel

**DOI:** 10.3390/mi7020033

**Published:** 2016-02-19

**Authors:** Huei Chu Weng

**Affiliations:** Department of Mechanical Engineering, Chung Yuan Christian University, Taoyuan 32023, Taiwan; hcweng@cycu.edu.tw; Tel.: +886-3-265-4311

**Keywords:** microfluidics, mixed convection, thermocreep convection, gas rarefaction, thermal creep

## Abstract

This study examines how thermal creep affects the mixed convection in a long horizontal parallel-plate microchannel under a pressure drop and a temperature rise. The analytical solutions of the fully developed thermal-flow fields and the corresponding characteristics are derived based on the Maxwell boundary conditions with thermal creep and presented for the physical properties of air at the standard reference state. The calculated thermal-flow characteristics reveal that thermal creep has an appreciable effect on the velocity slip, flow rate, and heat transfer rate but a negligible effect on the flow drag. Such a creep effect could be further magnified by decreasing the pressure drop or increasing the Knudsen number.

## 1. Introduction

Recent advances in microelectromechanical systems (MEMS) and nanotechnology have promoted a rapid development in mini/micro/nanoscale flow and heat transfer. The main topics include the flow and heat transfer influenced by micro/nanostructures (tissues, cells, crystal grains, nanoparticles, nanowires, gratings, *etc.*) and the flow and heat transfer at the mini/micro/nanoscale (in micro/nanochannels, micro/nanotubes, micro/nanoannuli, *etc.*). Recently, the flow and heat transfer issues of nanofluids (carry liquids with nanostructures) [1,2,3,4] and rarefied gases (gases in relatively low-density environments) [5,6,7,8] at the mini/microscale have arisen from reductions in the size of fluidic devices or their new applications in practice, e.g., microchip cooling, microheat exchanging, microelectrochemical cell transport, microreactor hydrogenation-reaction conduction [9,10,11,12]. The physical aspects in microfluidic devices may deviate from those presented at the macroscale. A fundamental microfluidic understanding is then required for technological applications. 

Gas rarefaction can be observed when fluidic devices get smaller. The rarefaction effect on physical aspects is characterized by the Knudsen number *Kn*. This dimensionless number is defined as the ratio of the molecular mean free path (the mean distance that a molecule travels between two consecutive collisions) to a characteristic geometric length. Schaaf and Chambré [13] suggested a classification system for a flow regime based on *Kn*. The flow for *Kn* ≤ 0.01 lies in the continuum regime, and the continuum hypothesis holds in this flow regime. The flow for 0.01 < *Kn* ≤ 0.1 lies in the slip regime, and the rarefaction effect may be noticeable and the continuum field equations subject to first-order slip (Maxwell) boundary conditions could be still valid in this flow regime [14,15,16,17,18]. Arkilic *et al.* [14], Chen and Weng [19], and Weng and Chen [20] originally studied the analytical investigations aimed at pressure-driven slip flow, buoyancy-driven slip flow, and thermocreep-driven slip flow at the microscale, respectively. Many theoretical studies over the past two decades have been further conducted on microscale slip-flow forced convection, natural convection, and mixed convection. For forced convection, Tunc and Bayazitoglu [21] analytically obtained a fully developed convection solution for an isoflux rectangular microchannel. Renksizbulut *et al.* [22] conducted a numerical study of the convection in the developing region of an isothermal rectangular microchannel. Aydin and Avci [23] analytically analyzed the fully developed convection in an isoflux/isothermal microtube considering the viscous dissipation effect. Avci and Aydin [24] analytically investigated the fully developed convection in a microannulus between an isoflux cylinder and an adiabatic cylinder. Shojaeian and Dibaji [25] numerically investigated the fully developed convection in an isothermal triangular microchannel. Sadeghi and Saidi [26] reported an analytical study of the viscous dissipation effect on the fully developed convection in a planar/annular microchannel with asymmetric wall heat fluxes. Çetin [27] modeled the fully developed convection in an isoflux planar/circular microchannel based on the second-order slip and local heat flux boundary conditions. Weng and Chen [28] developed a mathematical model of the fully developed magnetogas dynamic convection in an isothermal planar microchannel under an applied electric and magnetic field. Buonomo *et al.* [29] proposed an analytical solution for the fully developed convection in a microchannel filled with a porous medium under the local thermal non-equilibrium condition. Weng [30] and Weng and Liu [31] analytically solved the Navier-Stokes and energy equations subject to the second-order boundary conditions for the fully developed convection in an isoflux/isothermal planar microchannel. Wang *et al.* [32] analytically investigated the fully developed convection in a porous microtube under the local thermal non-equilibrium condition. As for natural convection, Chen and Weng [19] analytically studied the fully developed convection in a vertical planar microchannel with asymmetrically isothermal plates. Chen and Weng [33] numerically investigated the developing convection in an asymmetrically, isothermally heated planar microchannel. Haddad *et al.* [34] numerically modeled the developing convection in an isothermal planar microchannel filled with porous media. Biswal *et al.* [35] conducted a numerical study of the developing convection in an isothermal planar microchannel using the semi-implicit method for pressure-linked equations. Chakraborty *et al.* [36] performed a boundary layer integral analysis of the developing convection. Weng and Chen [37,38] examined the roles of variable physical properties and thermal creep in fully developed convection. Weng and Chen [39] conducted a study on the reduced flow drag and enhanced heat transfer over an asymmetrically, isothermally heated wall of a vertical annular microchannel. Buonomo and Manca [40] reported numerical solutions for the developing convection in a planar microchannel with asymmetric wall heat fluxes. Buonomo and Manca [41] further carried out an investigation of transient convection. Wang and Ng [42] looked into steady-state, fully developed convection in a planar microchannel with one wall exhibiting a superhydrophobic surface and another exhibiting a normal surface. Jha *et al.* [43] analytically investigated the fully developed convection in an annular microchannel with an asymmetrically isothermal porous cylinder. For mixed convection, Avci and Aydin [44] analytically studied the fully developed convection in an asymmetrically, isothermally heated vertical planar microchannel. Avci and Aydin [45,46] further considered the cases of convection between two isoflux walls and between two concentric microtubes. Weng and Jian [47] numerically examined the developing convection in an isothermal planar microchannel based on the second-order slip and jump boundary conditions. Jian and Weng [48] analytically examined the role of second-order slip in the fully developed convection through an asymmetrically, isothermally heated planar microchannel. Sadeghi and Baghani [49] investigated the fully developed convection in polygonal and rectangular microducts considering two axially constant heat flux boundary conditions. Akbulut [50] conducted an analysis of the entropy generation of the fully developed convection in a planar microchannel with asymmetric wall heat fluxes. In the literature, Çetin [27] and Weng and Chen [38] only investigated the influence of thermal creep due to constant wall heat fluxes, respectively, in forced and natural convection, and the previous mixed convection studies only investigated the combined forced and natural convection. The combined forced and thermocreep convection resulting from the pressure drop and temperature rise between duct entry and duct exit should be further studied.

Microfluidic devices with different entry and exit conditions are frequently encountered. In the present study, the combined forced and thermocreep convection through a long horizontal parallel-plate microchannel under a pressure drop and a temperature rise is studied. The Navier-Stokes and energy equations with the Maxwell boundary conditions considering thermal creep are first solved in an analytical way for the fully developed thermal-flow fields and the corresponding characteristics. The calculated results are then presented for the physical properties of air at the standard reference state. The influence of thermal creep on the thermal-flow characteristics with respect to the velocity slip, flow rate, flow drag, and heat transfer rate is further examined in detail and some conclusions are finally drawn. 

## 2. Basic Equations

### 2.1. Field Equations and Slip Conditions

**Figure 1:**
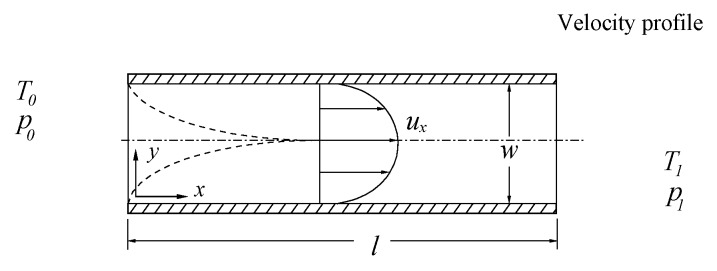
Model geometry.

Consider a horizontal parallel-plate microchannel with a uniform cross-section whose length *l* is very large compared to its width *w*, as shown in Figure 1. Assume that the flow in the microchannel is from a reservoir of gases of density *ρ*, shear viscosity *μ*, thermal expansion coefficient *β*, constant-pressure specific heat *c_p_*, and thermal conductivity *k* at a fixed pressure and temperature and it discharges to an area of lower pressure and higher temperature. It results in both a pressure drop and a temperature rise in the microchannel. Let *x* and *y* denote the horizontal and vertical coordinates, let *μ_x_* and *μ_y_* denote the x and y components of the velocity vector, let *T* be the temperature, and let *p* be the pressure. If we neglect the effects of flow compressibility (considering a low-speed gas microflow) [51] and variable thermophysical properties (considering a small temperature variation from the channel entry to the channel exit) [37], then the two-dimensional steady incompressible boundary layer equations for continuity, momentum, and energy are:

(1) $$\frac{\partial u_{x}}{\partial x}+\frac{\partial u_{y}}{\partial y}=0$$

(2) $$\rho_{0}\left(u_{x}\frac{\partial u_{x}}{\partial x}+u_{y}\frac{\partial u_{x}}{\partial y}\right)=-\frac{dp}{dx}+\mu_{0}\frac{\partial^{2}u_{x}}{\partial y^{2}}$$

(3) $$\rho_{0}c_{p0}\left(u_{x}\frac{\partial T}{\partial x}+u_{y}\frac{\partial T}{\partial y}\right)=k_{0}\frac{\partial^{2}T}{\partial y^{2}}+\mu_{0}{\left(\frac{\partial u_{x}}{\partial y}\right)}^{2}$$

where the subscripts 0 and 1 indicate the reservoir and discharge area values, respectively, and the subscript *i* indicates the inlet values. In Equation (2), the pressure gradient *–dp/dx* provides the driving force for the transport mechanism of forced convection. It should be noted that if the flow speed is low enough, the system does not significantly contribute to the volumetric dilatation rate (the rate of volume change per unit volume); that is, considering a low-speed fluid flow supports the neglect of flow compressibility [52]. The compressibility effect on the friction coefficient and Nusselt number of gas microflow has been numerically examined by Guo and Wu [51], and the inlet Mach number limits of the incompressible flow assumption were investigated in the literature.

The velocity and temperature boundary conditions with slip and jump resulting from gas rarefaction are on the basis of Maxwell’s expression and Smoluchowski’s expression, respectively [53,54]:

(4) $$u_{gw}=u_{w}\pm \frac{2-\sigma_{v}}{\sigma_{v}}\;\frac{1}{\rho {\left(2\hat{R}T_{gw}/\pi \right)}^{1/2}}\tau_{rt}+\frac{3}{4}\frac{\gamma -1}{\gamma }\frac{Pr}{\rho \hat{R}T_{gw}}\left(-q_{t}\right)$$

(5) $$T_{gw}=T_{w}\pm \frac{2-\sigma_{e}}{\sigma_{e}}\frac{2\left(\gamma -1\right)}{\gamma +1}\frac{1}{\hat{R}\rho {\left(2\hat{R}T_{gw}/\pi \right)}^{1/2}}\;\left(-q_{r}\right)$$

where the subscript *w* denotes the wall values, the subscript *gw* denotes the gas values near the wall surface, the subscripts r and t denote the gas values near the wall surface in the normal and tangential directions, respectively, *τ_ij_* is the stress tensor expressed in index notation, *q_i_* is the heat flux vector expressed in index notation, *σ_v_* is the tangential momentum accommodation coefficient, *σ_e_* is the thermal accommodation coefficient, *γ* is the ratio of specific heats, and $\hat{R}$ is the specific gas constant.

Assume that the hydrodynamically fully developed flow is achieved in the microchannel (considering a long microchannel and obeying the limit: $\partial u_{x}/\partial x=0$ and *μ_y_* = 0), then the field Equations (1)−(3) can be simplified as:

(6) $$\frac{dp}{dx}-\mu_{0}\frac{d^{2}u_{x}}{dy^{2}}=0$$

(7) $$\rho_{0}\;c_{p,0}\left(u_{x}\frac{\partial T}{\partial x}\right)=k_{0}\frac{\partial^{2}T}{\partial y^{2}}+\mu_{0}{\left(\frac{du_{x}}{dy}\right)}^{2}$$

The boundary conditions (4) and (5) can be written as:

(8) $$\begin{array}{l} u_{x}(\;0)=\frac{2-\sigma_{v}}{\sigma_{v}}\lambda \frac{du_{x}(0)}{dy}+\frac{3}{2\pi }\frac{\gamma -1}{\gamma }\frac{c_{p,0}\rho_{0}}{\mu_{0}}\lambda^{2}\frac{\partial T(x,\;0)}{\partial x}\\ u_{x}(\;w)=-\frac{2-\sigma_{v}}{\sigma_{v}}\lambda \frac{du_{x}(w)}{dy}+\frac{3}{2\pi }\frac{\gamma -1}{\gamma }\frac{c_{p,0}\rho_{0}}{\mu_{0}}\lambda^{2}\frac{\partial T(x,\;w)}{\partial x} \end{array}\}$$

Here, the rarefaction effect is related to the molecular mean free path *λ* by:

(9) $$\lambda =\frac{\sqrt{\pi \hat{R}T_{0}/2}\mu_{0}}{p_{0}}$$

For simplicity, here we only consider the Maxwell boundary conditions. The second terms in Equation (8) result from thermal creep and provide the driving force for the transport mechanism of thermocreep convection. Thermal creep is a phenomenon where gas molecules move from a cooler region towards a hotter region. Note that the wall temperature could be treated as a linear function due to the steady heat conduction behavior between two reservoirs with different temperatures.

### 2.2. Non-Dimensionalization

The model variables in Equations (6)−(8) can be nondimensionalized by introducing the following dimensionless variables and parameters:

(10) $$\begin{array}{l} X=\frac{x}{l_{c}Gr},\;Y=\frac{y}{l_{c}},\;U=\frac{u_{x}}{u_{c}},\Theta =\frac{T-T_{c}}{T_{1}-T_{0}},\;P=\frac{p}{p_{c}},\\ Gr=\frac{\rho_{0}^{2}g\beta_{0}\left(T_{1}-T_{0}\right)l_{c}^{3}}{\mu_{0}^{2}},\;Ec=\frac{u_{c}^{2}}{c_{p,0}\left(T_{1}-T_{0}\right)},\;Pr=\frac{c_{p,0}\mu_{0}}{k_{0}},\;Kn=\frac{\lambda }{l_{c}} \end{array}\}$$

where *g* is the gravitational acceleration. In Equation (10), *Gr*, *Ec*, *Pr*, and *Kn* are the dimensionless numbers for the problem and are known as the Grashof number, the Eckert number, the Prandtl number, and the Knudsen number, respectively, and *l_c_*, *μ_c_*, *T_c_*, and *p_c_* are the characteristic length, velocity, temperature, and pressure, respectively, and are defined as:

(11) $$l_{c}=w,\;u_{c}=\frac{\rho_{0}\;g\beta_{0}\left(T_{1}-T_{0}\right)l_{c}^{2}}{\mu_{0}},\;T_{c}=T_{0},\;p_{c}=\rho_{0}u_{c}^{2}$$

Thus, the dimensionless Navier-Stokes and energy equations are:

(12) $$\frac{dP}{dX}-\frac{d^{2}U}{dY^{2}}=0$$

(13) $$PrU\frac{\partial \Theta }{\partial X}=\frac{\partial^{2}\Theta }{\partial Y^{2}}+PrEc{\left(\frac{dU}{dY}\right)}^{2}$$

The dimensionless Maxwell boundary conditions are:

(14) $$\begin{array}{l} U(\;0)=\frac{2-\sigma_{v}}{\sigma_{v}}Kn\frac{dU(0)}{dY}+\frac{3}{2\pi }\frac{\gamma -1}{\gamma }\frac{1}{Ec}Kn^{2}\frac{\partial \Theta (X,\;0)}{\partial X}\\ U(\;1)=-\frac{2-\sigma_{v}}{\sigma_{v}}Kn\frac{dU(1)}{dY}+\frac{3}{2\pi }\frac{\gamma -1}{\gamma }\frac{1}{Ec}Kn^{2}\frac{\partial \Theta (X,\;1)}{\partial X} \end{array}\}$$

## 3. Analytical Solutions

The analytical solutions of the fully developed thermal-flow fields and the corresponding characteristics could be derived by solving the Navier-Stokes and energy equations (Equations (12) and (13)) subject to the Maxwell boundary conditions (Equation (14)). One can see from Equations (12) and (14) that a solution of the form *U(Y)* satisfying the momentum equation is possible only if we let both *dP/dX* and ∂Θ/∂X be constants, assuming *dP/dX = C_0_* and $\partial \Theta /\partial X=C_{1}$, respectively. Constant temperature gradient implies that the hydrodynamically fully developed flow is also thermally fully developed. The momentum equation (Equation (12)) is a second derivative with respect to *Y*, and the equation can then be integrated twice to give an expression for the dimensionless velocity as a function of *Y*:

(15) $$U(Y)=C_{3}+C_{2}Y+\frac{1}{2}C_{0}Y^{2}$$

An expression for the dimensionless temperature can be found by substituting Equation (15) into the energy equation (Equation (13)) and then by integrating it twice with respect to *Y* and once with respect to *X*:

(16) $$\begin{array}{ll} \Theta (X,\;Y)= & C_{5}+C_{4}Y+PrC_{1}\left(\frac{1}{2}C_{3}Y^{2}+\frac{1}{6}C_{2}Y^{3}+\frac{1}{24}C_{0}Y^{4}\right)\\ & -PrEc\left(\frac{1}{2}C_{2}^{2}Y^{2}+\frac{1}{3}C_{0}C_{2}Y^{3}+\frac{1}{12}C_{0}^{2}Y^{4}\right)+C_{1}X \end{array}$$

An expression for the dimensionless pressure can be obtained by integrating the pressure gradient *dP/dX = C_0_* once with respect to *X*:

(17) $$P(X)=C_{6}+C_{0}X+P_{0}$$

By applying the boundary conditions given in Equation (14), the symmetric condition ∂Θ(X, 1/2)/∂Y=0, and the open-end conditions *P(0) =P_0_ – M^2^/2* [33], *P(L) = P_1_*, Θ(0, 0)=0, and Θ(L, 0)=1, the six unknown constants can then be obtained as:

(18) $$\begin{array}{l} C_{0}=\frac{-\Delta P+{\dot{M}}^{2}/2}{L},\;C_{1}=\frac{1}{L},\\ C_{2}=-\frac{1}{2}C_{0},\;C_{3}=-\frac{1}{2}\beta_{v}C_{0}+\beta_{vc}C_{1},\\ C_{4}=\frac{Pr}{24}\left(\left(1+6\beta_{v}\right)C_{0}C_{1}-12\beta_{vc}C_{1}^{2}+EcC_{0}^{2}\right),\\ C_{5}=0,\;C_{6}=-\frac{1}{2}{\dot{M}}^{2} \end{array}\}$$

where

(19) $$\beta_{v}=\frac{2-\sigma_{v}}{\sigma_{v}}Kn,\;\beta_{vc}=\frac{3}{2\pi }\frac{\gamma -1}{\gamma }\frac{1}{Ec}Kn^{2},\;L=\frac{l}{l_{c}Gr},\;\dot{M}=\int_{0}^{1}UdY,\;\Delta P=P_{0}-P_{1}$$

The nondimensionalization of the wall shear stress $\tau_{w}$ as an average flow drag is:

(20) $$\bar{\Gamma }=\frac{\tau_{w}}{\mu_{0}u_{c}/l_{c}}=\frac{dU(0)}{dY}\;(\text{or }-\frac{dU(1)}{dY})=-\frac{1}{2}C_{0}$$

The nondimensionalization of the heat absorbed by gas from the entrance to the exit q as an average heat transfer rate is:

(21) $$\begin{array}{l} \bar{Nu}=\frac{\mu_{0}^{2}q}{2\rho_{0}^{2}g\beta_{0}k_{0}{\left(T_{1}-T_{0}\right)}^{2}l_{c}^{2}l}=\frac{c_{p,0}\mu_{0}^{2}\int_{0}^{w}u_{x}\left(T(l,\;y)-T_{c}\right)dy}{2\rho_{0}g\beta_{0}k_{0}{\left(T_{1}-T_{0}\right)}^{2}l_{c}^{2}l}\\ \;=\frac{Pr}{2GrL}\left(\begin{array}{l} -\frac{1}{168}PrEcC_{0}^{3}+\frac{1}{336}PrC_{0}^{2}C_{1}-\frac{1}{24}PrEcC_{0}^{2}C_{2}-\frac{1}{60}PrEcC_{0}^{2}C_{3}\\ +\frac{1}{48}PrC_{0}C_{1}C_{2}+\frac{7}{120}PrC_{0}C_{1}C_{3}-\frac{7}{60}PrEcC_{0}C_{2}^{2}-\frac{1}{12}PrEcC_{0}C_{2}C_{3}\\ +\frac{1}{30}PrC_{1}C_{2}^{2}+\frac{1}{6}PrC_{1}C_{2}C_{3}+\frac{1}{6}PrC_{1}C_{3}^{2}-\frac{1}{8}PrEcC_{2}^{3}\\ -\frac{1}{6}PrEcC_{2}^{2}C_{3}+\frac{1}{8}C_{0}C_{4}+\frac{1}{6}C_{0}C_{1}L+\frac{1}{3}C_{2}C_{4}+\frac{1}{2}C_{1}C_{2}L+\frac{1}{2}C_{3}C_{4}+C_{1}C_{3}L \end{array}\right) \end{array}$$

By using the flow-rate expression $\dot{M}=\int_{0}^{1}UdY$, the dimensionless channel length can then be expressed by:

(22) $$L=-\frac{1}{24}\left(1+6\beta_{v}\right)\left(\dot{M}-\frac{2\Delta P}{\dot{M}}\right)+\frac{\beta_{vc}}{\dot{M}}$$

Weng and Chen [20] have analytically studied the thermocreep convection in a long horizontal parallel-plate microchannel (without considering a pressure drop). If no pressure driving force is needed, the present analytical solutions, Equations (15)−(17), (20) and (21), are identical to the expressions obtained by Weng and Chen. 

## 4. Results and Discussion

The calculated results for the fully developed thermal-flow fields, Equations (15)−(17), and the corresponding characteristics, Equations (20) and (21), are presented for the physical properties of air at the standard reference state (25 °C and 1 atm). The influence of thermal creep on the combined forced and thermocreep convection is then examined and some conclusions are finally drawn. Assume that the flow in the microchannel is from a reservoir of air of the molecular mean free path *λ* = 0.666 × 10^−7^ m, density *ρ*_0_ = 1.185 kg/m^3^, shear viscosity *μ*_0_ = 1.842 × 10^−5^ kg/m·s, thermal expansion coefficient *β*_0_ = 3.35 × 10^−3^ 1/K, constant-pressure specific heat *c_p,0_*= 1007 J/kg·K, thermal conductivity *k*_0_ = 2.61 × 10^−2^ W/m·K, and specific heat ratio *γ* = 1.399 at the standard reference state. The parametric study is then performed with Knudsen numbers up to 0.1, meaning gas flows in the continuum and slip flow regimes. The corresponding channel width is greater than 0.666 μm. It has been shown by three-dimensional (3D) stereoscopic particle image velocimetry/particle tracking velocimetry (PIV/PTV) [55] that the predictions of mathematical analysis match experimental data to within 3%, which is within the experimental error of the fabrication of the geometry, and therefore there is no need to validate transport theory. Also, previous investigations [14,15,16,17,18] have concluded that the continuum field equations subject to first-order slip boundary conditions could be valid in these flow regimes. In addition to *Kn*, the ranges of the parameters *L*, *Ec*, and Δ*P* are restricted to *l/w* = *GrL ≥ 100*, *T*_1_ – *T*_0_ ≤ 30 K, and *p*_0_ – *p*_1_ = *p*_c_Δ*P* ≤ 10 kPa, respectively, so as to ensure that the required assumptions are satisfied. It should be noted that for simplicity, the reported results have been conducted for complete accommodation (*i.e.*, *σ_v_* = 1).

**Figure 2:**
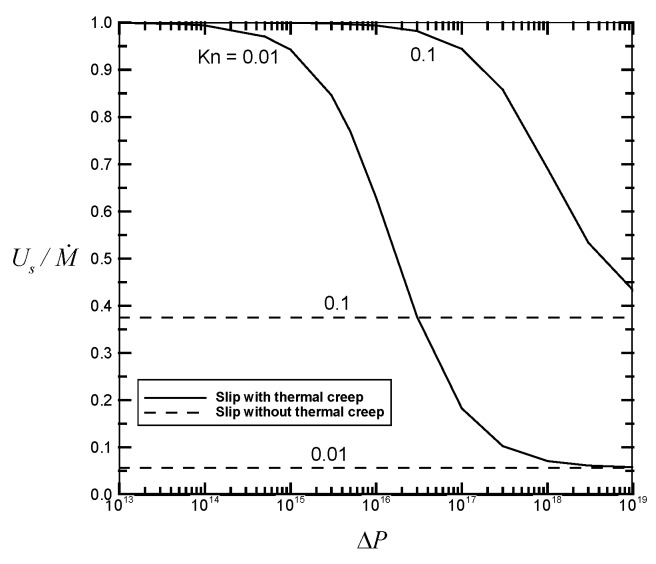
Velocity slip $U_{s}/\dot{M}$ *versus* the dimensionless pressure drop Δ*P* for different values of *Kn* with *GrL* = 10^2^ and *Ec* = 10^−20^.

**Figure 3:**
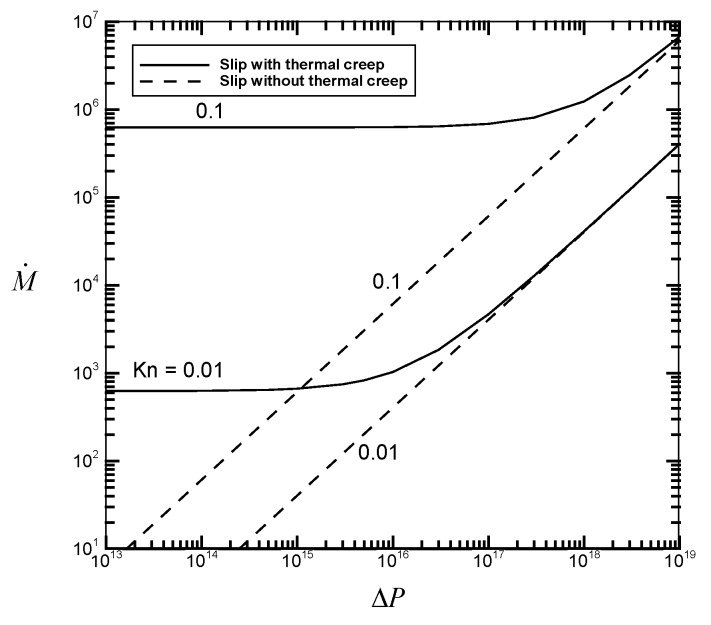
Flow rate $\dot{M}$ *versus* the dimensionless pressure drop Δ*P* for different values of *Kn* with *GrL* = 10^2^ and *Ec* = 10^−20^.

**Figure 4:**
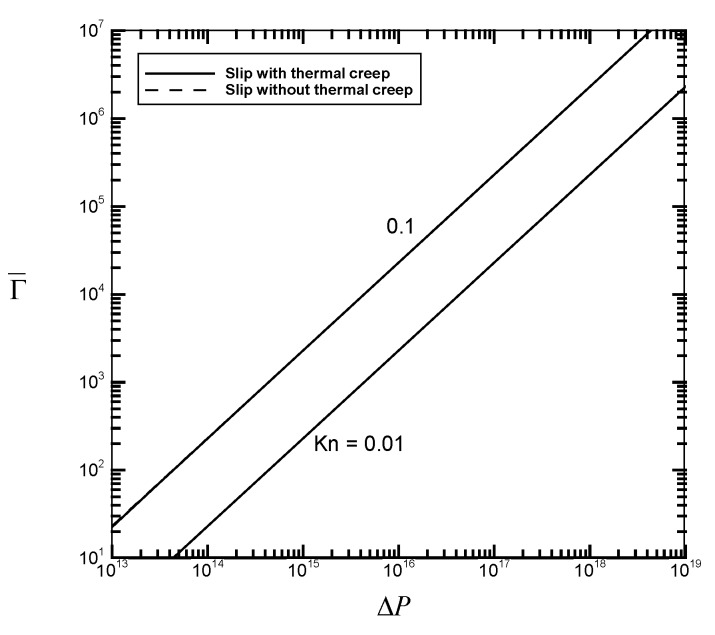
Average flow drag $\bar{\Gamma }$ *versus* the dimensionless pressure drop Δ*P* for different values of *Kn* with *GrL* = 10^2^ and *Ec* = 10^−20^.

**Figure 5:**
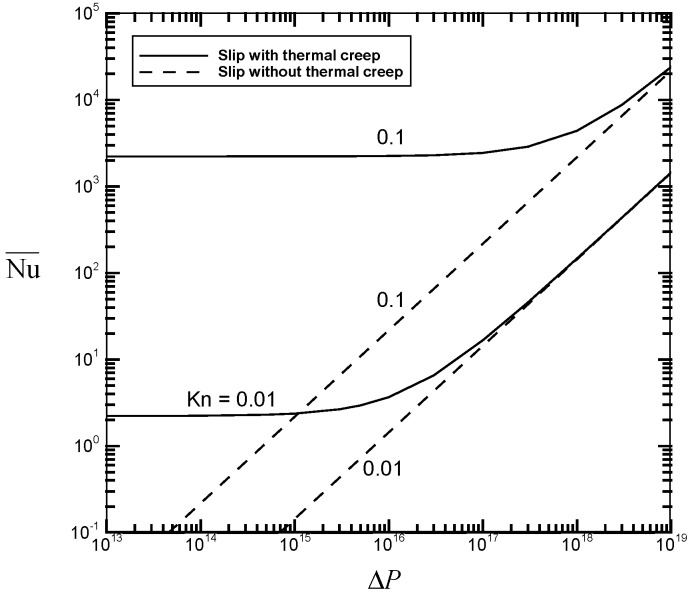
Average heat transfer rate $\bar{Nu}$ *versus* the dimensionless pressure drop Δ*P* for different values of *Kn* with *GrL* = 10^2^ and *Ec* = 10^−20^.

Figure 2, Figure 3, Figure 4 and Figure 5 illustrate the variations of the velocity slip $U_{s}/\dot{M}$, the flow rate $\dot{M}$, the average flow drag $\bar{\Gamma }$, and the average heat transfer rate $\bar{Nu}$ as a function of the pressure drop Δ*P* for different values of the Knudsen number *Kn* with fixed values of the channel length *GrL* and the Eckert number *Ec*. Thermal creep, providing the driving force for the transport mechanism of thermocreep convection, could speed up the fluid motion near the walls and then results in an additional velocity slip. From Figure 2, it is found from the comparison between the solid line (slip with thermal creep) and the dashed line (slip without thermal creep) that the velocity slip phenomenon due to thermal creep decreases with the pressure drop Δ*P* and could be eventually negligible for large-pressure-driven flows. This means that when forced convection is dominant, the effect of thermal creep could be neglected. However, when the Knudsen number *Kn* increases from 0.01 to 0.1, it leads to the difficulty of neglecting the creep effect. It implies that thermocreep convection could play a more important role in microfluidic devices. From Figure 3, Figure 4 and Figure 5, it is observed that the effect of thermal creep (resulting in an additional velocity slip) could be negligible on the flow drag but significant on the flow rate and heat transfer rate. The flow rate and heat transfer rate are found to be enlarged due to the thermocreep driving mechanism, which keeps the same shear stress on the wall surface. Such a creep effect could be further magnified by decreasing the pressure drop or increasing the Knudsen number.

## 5. Conclusions

Nowadays, microelectromechanical systems (MEMS) have led to reductions in the size of fluidic devices. When a fluidic device gets smaller down to the microscale, the flow and heat transfer become a more important issue. There is practical interest in investigating the thermal creep phenomenon since most microfluidic devices in engineering are non-isothermal. In this paper, a study on the modeling of combined forced and thermocreep convection in a long horizontal parallel-plate microchannel has been made by analytically solving the Navier-Stokes and energy equations with the Maxwell boundary conditions. The influence of thermal creep on the thermal-flow characteristics with respect to the velocity slip, flow rate, flow drag, and heat transfer rate were investigated for the physical properties of air at the standard reference state. The calculated fully developed results were proven to have negligible creep effect on the wall friction. However, thermal creep due to temperature rise along the wall surface could speed up the velocity near the walls and enlarge the flow and heat transfer rates. As the pressure drop decreases or the Knudsen number increases, the creep effect could be further enhanced.

The results help us to understand the rarefied gas transport behavior in non-isothermal microfluidic devices with different open-end conditions (inlet and outlet pressures and temperatures) and benefit the designs of microfluidic devices in need of enhanced flow and heat transfer rates.

## Abbreviations

*c_p_*	specific heat at constant pressure (J/kg·K)
*Ec*	Ecker number
*g*	gravitational acceleration (m/s^2^)
*Gr*	Grashof number
*K*	thermal conductivity (W/m·K)
*Kn*	Knudsen number
*l*	channel length (m)
*L*	dimensionless channel length
$\dot{M}$	dimensionless volume flow rate
$\bar{Nu}$	dimensionless average heat transfer rate
*p*	pressure (Pa)
*P*	dimensionless pressure
ΔP	dimensionless pressure drop
*Pr*	Prandtl number
q	heat absorbed by fluid between channel entry and channel exit (W/m)
*q_i_*	heat flux vector expressed in index notation (W/m)
$\hat{R}$	specific gas constant (m^2^/K·s^2^)
*T*	temperature (K)
*μ_x_, μ_y_*	velocity components in *x*, *y* directions (m/s)
*U*	dimensionless velocity
*Us*	dimensionless velocity slip
*w*	channel width (m)
*x,y*	rectangular coordinate system (m)
*X,Y*	dimensionless rectangular coordinate system
*β*	thermal expansion coefficient (1/K)
*γ*	specific heat ratio
$\bar{\Gamma }$	dimensionless average flow drag
*λ*	molecular mean free path (m)
Θ	dimensionless temperature
*μ*	shear viscosity (kg/m·s)
*ρ*	density (kg/m^3^)
*Σ_e_*	thermal accommodation coefficient
*σ_v_*	tangential momentum accommodation coefficient
*τ_ij_*	stress tensor expressed in index notation (Pa)
*τ_w_*	flow drag on wall surface per unit area (Pa)
*c*	characteristic values
*gw*	gas values near the wall surface
*i*	inlet values
*r, t*	gas values near the wall surface in the normal and tangential directions, respectively
*w*	wall values
*0, 1*	reservoir and discharge-area values, respectively
